# Articular Contact Mechanics from an Asymptotic Modeling Perspective: A Review

**DOI:** 10.3389/fbioe.2016.00083

**Published:** 2016-11-01

**Authors:** Ivan Argatov, Gennady Mishuris

**Affiliations:** ^1^Institut für Mechanik, Technische Universität Berlin, Berlin, Germany; ^2^Institute of Mathematics and Physics, Aberystwyth University, Ceredigion, UK

**Keywords:** articular contact, knee joint, articular cartilage, asymptotic model, thin layer, biphasic theory, deformation, damage

## Abstract

In the present paper, we review the current state-of-the-art in asymptotic modeling of articular contact. Particular attention has been given to the knee joint contact mechanics with a special emphasis on implications drawn from the asymptotic models, including average characteristics for articular cartilage layer. By listing a number of complicating effects such as transverse anisotropy, non-homogeneity, variable thickness, nonlinear deformations, shear loading, and bone deformation, which may be accounted for by asymptotic modeling, some unsolved problems and directions for future research are also discussed.

## 1 Introduction

Articular cartilage is a non-vascular soft tissue, which covers the ends of bones and thereby prevents damage in their contact. In the knee joint, a half of body weight is transferred through the articular contact in a quiet standing position, and the level of loading increases progressively in walking, running, and jumping. Experimental investigations (van den Bogert et al., 1999) have shown that joint contact forces of up to 300% body weight can occur even during normal walking, and may rise to 550% during the push-off phase of running, whereas various skiing activities produce a joint contact force ranging from 400% body weight (long turns and flat slope) to 900% body weight during short turns on a steep slope.

The mechanical aspects of articular contact, such as the contact pressure pattern [e.g., which is changed due to some gait disorders (Rosneck et al., 2007)], the maximum level of loading, or the type of loading [e.g., impact (Herzog and Federico, 2006; Kessler et al., 2006; Garcia et al., 2008)] are closely related to he development and progression of osteoarthritis (Maly et al., 2008). On the other hand, the analytical modeling of articular contact is necessary in formulating equations for the reaction forces generated in joints during multibody simulations of human and animal movements (Delp and Loan, 2000; Machado et al., 2011).

With a tremendous progress in development of computer simulation tools, the early analytical models of articular contact (Eberhardt et al., 1990; Blankevoort et al., 1991; Eberhard et al., 1999; Bei and Fregly, 2004) were succeeded by FE models (Wu et al., 1997; Caruntu and Hefzy, 2004; Wilson et al., 2005a; Galbusera et al., 2014) that have been steadily improved in accuracy and realistic presentation of the contacting parts (Caruntu and Hefzy, 2004) as well as their complexity has been increased by accounting for many factors such as microstructure (Bursać et al., 2000), meniscus (Peña et al., 2006), fluid exudation (Carter et al., 2004), which are usually neglected in analytical studies.

Asymptotic modeling is a mathematical modeling approach aimed to simplify a given mathematical model by considering the so-called limit situation with respect to a certain dimensionless parameter. As a result of asymptotic modeling, we obtain an asymptotic model, which bears the main features of the original mathematical model essential for the chosen limit situation (Argatov, 2012a).

## 2 Asymptotic Modeling of Articular Contact

### 2.1 Asymptotic Model for Deformation of Articular Cartilage

It is well known (Poole et al., 2001) that articular cartilage mainly consists of extracellular matrix and interstitial fluid. Namely, this biphasic nature of cartilage was reflected in the mathematical model for its deformation response developed by Mow et al. (1980). In order to describe the deformation response of articular cartilage layer under an external load, a number of modeling issues should be fixed (including, the geometry of cartilage layer, the method of its loading, and the boundary conditions imposed at the layer surfaces).

**Figure 1:**
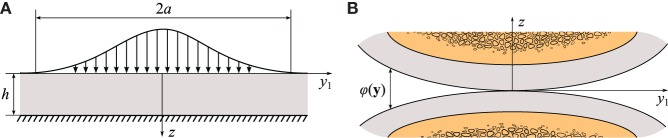
**(A)** Model problem for the deformation of articular cartilage layer [after Ateshian et al. (1994)]; **(B)** schematics of the initial contact geometry between two cartilage layers bonded to subchondral bones.

Figure 1A shows a biphasic layer bonded to an impermeable rigid base and loaded by an axisymmetric distributed normal load, which, for the sake of simplicity, does not change in time. Evidently, the problem contains a dimensionless parameter of geometrical nature *ε* = *h*/*a*, where *h* is the layer thickness and *a* is the radius of the loaded area. Under the assumption that *ε* ≪ 1, it becomes possible to look for the solution in the limit situation as *ε* → 0 in the form of a series with respect to the parameter *ε* by applying the corresponding perturbation technique. In this way, an asymptotic solution of the axisymmetric deformation problem for an isotropic biphasic layer was obtained by Ateshian et al. (1994).

It should be emphasized that articular cartilage can be regarded as a time-dependent material. So that its response to a suddenly applied normal pressure exhibits two limit situations in time: namely, the short-time response and the long-time (equilibrium) response, and it is interesting to observe (Ateshian et al., 1994; Barry and Holmes, 2001) that the instantaneous response of a biphasic tissue corresponds to that of an incompressible elastic material, whereas in the long-time regime (in the equilibrium state), the biphasic layer responds as a compressible material.

The axisymmetric asymptotic model by Ateshian et al. (1994) and Wu et al. (1996) was generalized for non-axisymmetric loading configurations (Argatov and Mishuris, 2011a) and extended to the cases of a transversely isotropic biphasic/viscoelastic layer (Argatov and Mishuris, 2011c, 2015b) and of a thin biphasic poroviscoelastic layer (Argatov and Mishuris, 2015c).

In particular, the first-order asymptotic solution for the normal displacements, *w*_0_(*t*, **y**), of the surface points of a bonded thin biphasic layer is obtained in the form

(1) $$w_{0}\left(t,\text{y}\right)=-\frac{h^{3}}{3G^{\prime }}\Delta_{y}p\left(t,\text{y}\right)-hk_{1}\overset{t}{\underset{0}{\int }}\;\Delta_{y}p\left(\tau ,\text{y}\right)\;d\tau .$$

Here, *p*(*t*, **y**) is a distributed surface load, which depends on the time variable *t* and the Cartesian coordinates **y** = (*y*_1_, *y*_2_) on the layer surface, $\Delta_{y}=\partial^{2}∕\partial y_{1}^{2}+\partial^{2}∕\partial y_{2}^{2}$ is the Laplace differential operator, *G*′ is the out-of-plane shear modulus of the solid matrix, *k*_1_ is the transverse (in-plane) permeability.

### 2.2 Contact Problem Formulation

In order to formulate the articular contact problem, it is first necessary to list the key mechanical quantities that play a major role in the contact phenomena. First of all, this is the pair of the contact force, *F*(*t*), and the contact approach, *δ*_0_(*t*), both being functions of time. The contact force *F* represents the total of external load, which is transferred through the joint, while *δ*_0_ represents the corresponding so-called generalized displacement and has a meaning of the normal displacement between the bones (whose deformation is usually neglected). Second, this is the pair of the contact pressure, *p*, and the layer deformations represented by the surface normal displacements, $w_{0}^{\left(1\right)}$ and $w_{0}^{\left(2\right)}$. The latter quantities can be regarded as internal variables (there is no way of non-invasive measuring the contact pressures in an intact joint).

Further, another important aspect of contact interactions between the cartilage layers is determined by the geometry of the layers, which, in turn, determines the gap function, *φ*(*y*_1_, *y*_2_) (see Figure 1B). The case of subchondral bones shaped as elliptic paraboloids [a commonly assumed geometry in the Hertzian theory of elastic contact (Johnson, 1985)] can be regarded as the main approximation for the tibiofemoral contact in the weight-bearing region [in particular, it covers the case of spherical bones assumed by Ateshian et al. (1994) and Wu et al. (1996)] and was introduced by Koo and Andriacchi (2007).

Thus, taking into account the contact condition of non-penetration inside the contact area, *ω*, that is

(2) $$w_{0}^{\left(1\right)}\left(t,\text{y}\right)+w_{0}^{\left(2\right)}\left(t,\text{y}\right)=\delta_{0}\left(t\right)-\varphi \left(\text{y}\right),\;\text{y}\in \omega \left(t\right),$$

and equation (1), which relates $w_{0}^{\left(n\right)}\left(t,\text{y}\right)$, *n* = 1, 2, to the contact pressure *p*(*t*, **y**), we arrive at the equation

(3) $$\Delta_{y}p\left(t,\text{y}\right)+\chi \overset{t}{\underset{0}{\int }}\;\Delta_{y}p\left(\tau ,\text{y}\right)\;d\tau =m\left(\varphi \left(\text{y}\right)-\delta_{0}\left(t\right)\right),$$

where the coefficients *χ* and *m* are given by

$$\chi =3\;\left(\frac{G_{1}^{\prime }k_{1}^{\left(1\right)}}{h_{1}^{2}}+\frac{G_{2}^{\prime }k_{1}^{\left(2\right)}}{h_{2}^{2}}\right)\;,\;m=3{\left(\frac{h_{1}^{3}}{G_{1}^{\prime }}+\frac{h_{2}^{3}}{G_{2}^{\prime }}\right)}^{\;-1}.$$

Of course, the approximate (asymptotic) mathematical model [equation (3)] is restricted to the short-time contact period, thereby may be oversimplifying many aspects of articular contact (some of them will be discussed later), including the assumption of direct contact between the cartilage layers without taking into account the influence of meniscus or interstitial fluid [see, e.g., the discussion given by Ateshian et al. (1994)].

### 2.3 Effect of Boundary Conditions

First of all, we emphasize that the contact area *ω* is not known in advance and depends on the value of the contact force

(4) $$F\left(t\right)=\underset{\omega \left(t\right)}{\iint }\;p\left(t,\text{y}\right)\;d\text{y}.$$

In order to be able to solve equation (3) uniquely, it is necessary to formulate two boundary conditions on the contour Γ(*t*) of the domain *ω*(*t*). One condition is obvious and follows from the continuity of the contact pressure, which is absent outside the contact area, i.e.,

(5) p(t,y)=0,y∈Γ(t),

Concerning another boundary condition

(6) $$\frac{\mathrm{\partial p}}{\mathrm{\partial n}}\left(t,\text{y}\right)=0,\;\text{y}\in \Gamma \left(t\right),$$

where ∂/∂*n* is the normal derivative, in the literature, there was a discussion (Hlaváček, 1999; Wu and Herzog, 2000; Argatov et al., 2016a).

Apart from the fact that the mathematical model [equations (3), (5), and (6)] incorporates the model for instantaneous response, it was shown (Argatov and Mishuris, 2015b) that the boundary conditions [equations (5) and (6)] are asymptotically exact for thin incompressible elastic layer in unilateral contact. At the same time, the question of imposing refined boundary conditions [like those introduced by Hlaváček (1999) in the axisymmetric case] is still open and requires the study of the corresponding boundary layer problem for a 2D biphasic strip.

On the other hand, when formulating the contact problem, a refined contact condition [instead of equation (2)] can be used, as shown by Mishuris and Argatov (2009) and Argatov and Mishuris (2010) in the axisymmetric case. The refined condition takes into account the tangential displacements, which undergo the contacting points during the contact deformation, thereby increasing the complexity of the contact problem in the non-axisymmetric case (Rogosin et al., 2016) and introducing a certain correction into the solution (namely, the relation between the contact force and the contact approach turns out to be most susceptible to the effect of tangential displacements).

### 2.4 Some Implications Drawn from the Asymptotic Models

Simple as it is, the asymptotic model [equations (3)–(6)], as applied in the axisymmetric case by Wu et al. (2000), sheds light on the influence of the degenerative changes in the articular cartilage mechanical properties on the contact pressure distribution. In particular, it is known (Korhonen et al., 2002) that the articular cartilage superficial zone, which is characterized by tangentially oriented collagen fibrils, is important for the deformation response of the articular cartilage layer. The effect of superficial zone was recently modeled (Argatov and Mishuris, 2016) by an extensible membrane coating attached to the surface of a thin bonded incompressible elastic layer, and it was shown that the reinforcing effect reduces the out-of-plane shear compliance of the elastic layer up to a maximum of four times (in the limit situation of an inextensible membrane).

When the asymptotic model for deformation of a thin biphasic layer [equation (1)] was generalized for a transversely isotropic layer (Argatov and Mishuris, 2015b), it highlighted the roles played by the transverse shear modulus *G*′ and the in-plane permeability *k*_1_. Recall that, while the shear modulus, *G*, for an isotropic material is related to its Young’s modulus, *E*, and Poisson’s ratio, *v*, *via* the formula *G* = *E*/[2(1 + *v*)], in the case of a transversely isotopic material *G*′ represents a material property independent from those measured in the confined and unconfined compression tests.

### 2.5 Average Characteristics for Articular Cartilage Layer

It is known that articular cartilage layers are inhomogeneous, anisotropic, non-uniform, and non-flat (Schinagl et al., 1997; Mow and Guo, 2002). At the same time, equations (1) and (3) operate with constant characteristics *h*, *G*′, *k*_1_, *R*_1_ and *R*_2_. The question of the model sensitivity with respect to the parameter variations was studied in a number of papers (Anderson et al., 2010; Argatov and Mishuris, 2011b; Argatov, 2013a). In particular, in the case of a thin transversely isotropic and transversely homogeneous (TITH) elastic layer, the average transverse shear modulus, ${\bar{G}}^{\prime }$, is given by the following formula (Argatov and Mishuris, 2015b):

$${\bar{G}}^{\prime }={\left(\frac{3}{h^{3}}\overset{h}{\underset{0}{\int }}\;\frac{z^{2}\text{dz}}{G^{\prime }\left(z\right)}\right)}^{\;-1}.$$

At the same time, the average thickness and curvature radii of the gap function depend on the extend of the contact area, over which the averaging is performed (Argatov, 2012a).

### 2.6 Contact Force Modeling for Multibody Simulations

There is a vast literature on modeling of reaction contact forces generated in joints (Silva et al., 1997; Flores et al., 2011; Machado et al., 2011; Monteiro et al., 2011). A majority of the employed models represent variations of the following model introduced by Hunt and Crossley, 1975:

(7) $$F=\text{bk}x^{n}\dot{x}+kx^{n}.$$

Here, *n* is a real constant, *k* is a stiffness coefficient, and *b* is a damping parameter.

A peculiarity of the force–displacement relation [equation (7)] is that the value of the force *F* returns to zero, when the displacement value *x* does the same. In biomechanical applications, equation (7) was used in a number of papers (Silva et al., 1997; Guess et al., 2010; Machado et al., 2010).

At the same time, the contact force model, which is based on the asymptotic model [equations (3)–(6)], shows a residual deformation, when the contact force vanishes. The same phenomenon is seen in viscoelastic models even for such simple as Maxwell and Kelvin–Voigt models (Argatov, 2013b; Argatov et al., 2016b).

However, it is known (Selyutina et al., 2015) that in the case of cyclic dynamic loading of a viscoelastic solid, the steady-state response will be analogous to that of the Hunt–Crossley model [equation (7)]. Therefore, by considering a steady-state response of the asymptotic model, we have established a link between the two models and expressed the coefficients *k* and *b* in equation (7) in terms of the biphasic layer parameters.

### 2.7 Damage and Fracture Criteria

It goes without saying that the damage and fracture phenomena in articular cartilage, which occur under loads exceeding the physical level (Aspden et al., 2002), are too complicated (Peña, 2011) to be captured by such simple analytical models as equations (3)–(6).

However, the simple analysis turns out to be very useful for identifying (Argatov and Mishuris, 2015a,d) the modeling framework of the laboratory impact tests (Jeffrey et al., 1995; Varga et al., 2007). Indeed, though the damage and fracture processes are multiscale, they are governed by the level of external loading during the impact event, whose evolution can be monitored at the macros-scale. In particular, by inspecting the coefficient of restitution, one can estimate the share of the impact energy dissipated or spent on the damage accumulation and surface fissuring or formation of small cracks.

## 3 Unsolved Problems and Directions for Future Research

### 3.1 Nonlinearity

Articular cartilage is a soft tissue and may undergo (moderately) large deformations without damage (Quinn et al., 2001; Morel and Quinn, 2004). However, the asymptotic model [equations (3)–(6)] was developed in the framework of the linear biphasic theory, and thus, its extension to the case of deformations, which cannot be regarded as small, will be very useful. Here, it is worth mentioning the known dependency of the cartilage permeability on the volumetric strain (Mow et al., 1980), which also was not accounted for by the linear asymptotic models, while this effect dumps the deformation very quickly and, thereby, making the fitting of experiments by asymptotic models quite difficult. It seems, furthermore, that Soltz and Ateshian (2000) obtain excellent results both in tension and compression by adopting a conewise linear elasticity (Curnier et al., 1995).

### 3.2 Compound Asymptotic Model for Merging the Short- and Long-Time Responses

Recall that the asymptotic model [equations (3)–(6)] was developed to capture the short-time asymptotics (Ateshian et al., 1994), and it leads to unrealistic predictions as *t* → ∞ (in particular, the contact approach is unbounded). On the other hand, the equilibrium response of the biphasic layer can be modeled by that of a compressible elastic layer. Thus, there are two asymptotic models, which could be merged into the so-called compound asymptotic model.

### 3.3 Triphasic Model

As a generalization of the biphasic theory for articular cartilage (Mow et al., 1980), the so-called triphasic theory, which combines the biphasic theory with the physico-chemical theory for ionic and polyionic solutions, has been developed by Lai et al. (1991). There is an undoubted interest in formulating the deformation problem for a thin triphasic layer and constructing its first-order asymptotic solution.

### 3.4 Meniscus

From a geometrical point of view, articular cartilage can be modeled as a layer (of variable thickness). A meniscus has a more complicated geometry (Peña et al., 2006), and, to the best of the authors’ knowledge, there is a lack of a simple analytical (approximate or asymptotic) model for the deformation response of menisci. Consequently, a generalization of the asymptotic model [equations (3)–(6)], which incorporates the meniscus deformation, will be useful, since the menisci transfer a significant proportion of the load across the knee joint (Fahmy et al., 1983).

### 3.5 Migrating Contact

During the cycle of loading–unloading in walking or running, the contact area between the articular cartilage layers changes and moves (Iseki and Tomatsu, 1976). Therefore, the so-called problem of migrating contact can be formulated (Chen et al., 2009; Argatov, 2012b). In the framework of the asymptotic model [equations (3)–(6)], such a problem was considered and an approximate solution was given for the steady-state regime. However, the corresponding problem of migrating contact for a viscoelastic/biphasic layer bonded to a rigid sphere periodically moving with rotation over the surface of another viscoelastic/biphasic layer bonded to a rigid flat base has not been investigated, yet even in the small thickness approximation.

### 3.6 Curved Layer Model

In particular, for the hip joint, the case of spherical geometry of the articular cartilage layer is very important. The corresponding contact was studied in the thin layer approximation (Argatov, 2011). Also, an approximate analysis of the deformation problem for a hemispherical biphasic layer was attempted recently by Quinonez et al. (2011). Note that, in the case of a curved compressible elastic layer, the first-order asymptotic theory was developed by Mal’kov (1998). However, the generalization of the asymptotic model [equation (1)] for a thin biphasic layer bonded to a rigid base shaped as an elliptic paraboloid is absent, and, correspondingly, the generalization of the asymptotic model [equations (3)–(6)], which takes into account the effect of the cartilage layer curvature is still missing. On the other hand, there is one more interesting outlook in this specific topic. What happens when there are two contact areas, which may interact? Looking at MR images of the ankle joint (Li et al., 2008), it seems quite a common situation that two contact areas merge under *in vivo* loading conditions.

### 3.7 Bone Deformation

The asymptotic model [equations (3)–(6)] neglects the deformation of the subchondral bones. However, for the case of intensive loading of the joint, the deformation of the bones may contribute to the contact pressure pattern. This effect was not analytically studied yet, though in the FE simulations usually (Anderson et al., 2010; Duarte et al., 2015) the bones are assumed to be compliant with relatively large elastic modulus compared to the elastic modulus of the articular cartilage layers. Here, it should be noted that under the dynamic loading (Laasanen et al., 2003; Park et al., 2004), the so-called dynamic elastic modulus of cartilage is much higher than that measured under quasi-static conditions.

### 3.8 Synovial Fluid Effect

The asymptotic model [equations (3)–(6)] assumes direct contact between the two cartilage layers, which can occur after some time when the synovial fluid is squeezed out of the contact region (Ateshian et al., 1994). The problem of squeezing of the synovial fluid was studied in a number of papers (Hou et al., 1992; Ruggiero et al., 2011; Yousfi et al., 2013). Of considerable practical interest is a generalization of the asymptotic model [equations (3)–(6)] that accounts for the synovial fluid effect in non-axisymmetric configuration. One concern is about the exudation of the interstitial fluid out from under the cartilage layers contact area (Caligaris and Ateshian, 2008). How important is the effect of the migrating boundaries on the fluid pressurization, and what boundary conditions should be imposed with respect to the contact pressure distribution?

### 3.9 Damage Accumulation and Impact-Induced Fissuring

The deformation problem for a biphasic layer (Figure 1A) was considered under quasi-static loading (Ateshian et al., 1994) and though neglecting the inertia effect, it can be applied to study the contact between cartilage layers under dynamic loading (Wu et al., 1998; Quinn et al., 2001) and impact loading (Jeffrey et al., 1995; Ewers et al., 2001) under normal physiological conditions (Aspden et al., 2002). As a first approximation, it was suggested (Argatov and Mishuris, 2015a) that the asymptotic model [equations (3)–(6)] can predict the deformation of articular cartilage and the damage accumulation process until the fracture moment. Of course, the further development of mathematical models for impact-induced fissuring (Atkinson et al., 1998; Kafka, 2002) will require a more sophisticated mathematical modeling framework [see, e.g., Peña (2011) and Mengoni and Ponthot (2015)]. Nevertheless, this simple model reveals the key model parameters, which should be reported in the experimental studies in order to facilitate the comparison between different experiments.

### 3.10 Shear Loading

The asymptotic model [equations (3)–(6)] considers the case of unilateral normal frictionless contact and is based on the asymptotic solution [equation (1)] of the deformation problem for a biphasic layer loaded by a normal distributed load. Due to a very small coefficient of friction for articular cartilage layers in contact *via* a film of synovial fluid, the tangential stresses are usually neglected in evaluation of the stress–strain state of the joint in physiologically normal conditions (Ateshian, 2009). However, under severe loading, e.g., in traumatic situations in sport, the cartilage layers can transform a significant shear loading (Carter and Wong, 1988). Therefore, the problem of tangential loading of a thin biphasic layer requires a special attention.

### 3.11 Non-Homogeneity

As it is known (Poole et al., 2001), articular cartilage is a non-homogeneous tissue with properties primarily varying with depth (Schinagl et al., 1997). Recently, the problem of normal loading of a thin biphasic layer was studied by (Vitucci et al., 2016) for a special case of exponential type of non-homogeneity. The obtained asymptotic solution can be used for generalizing the asymptotic model of uniateral contact [equations (3)–(6)] for this case (Vitucci and Mishuris, 2016).

### 3.12 Whole Joint Analytical Model

It is a very difficult problem to create a system of analytical models (let us say, sub-models), which account for the major mechanical aspects of articular contact in the knee joint, e.g., including the deformation of patella, menisci, and ligaments (Maquet, 1976). The aim of such asymptotic mathematical modeling is to predict (at least by the order of magnitude) the contact forces and the deformation factors in the joint for a range of physiological displacements of the bones.

### 3.13 Fibril-Reinforced Material Models

By accounting for the microstructure of articular cartilage, in the literature, a number of advanced material models for describing the deformation of cartilage have been presented (Korhonen et al., 2003; Wilson et al., 2005b; Freutel et al., 2014). Since the use of these models for analytical solution is confronted with considerable mathematical difficulties, it makes sense to solve the model deformation problem (Figure 1A) for a relatively thin fibril-reinforced layer by FE methods and highlighting the differences with the basic homogeneous case first studied by Armstrong (1986) and Ateshian et al. (1994) using an analytical technique.

### 3.14 FEM-Based Surrogate Models

It goes without saying that the articular cartilage contact problem with realistic geometry and that takes into account the effects (discussed above) represents a challenge for a real-time computer simulations of the knee joint dynamics in real activities such as the gait cycle (Pérez-González et al., 2008). In special cases, e.g., under the assumption of cyclic dynamic loading during walking when the contact loading configuration is repeated, the result of certain blocks of the computational algorithm can be fitted with computationally cheap surrogate contact analytical models. Such an approach, introduced by Lin et al. (2010), is called surrogate modeling. It is foreseen that the asymptotic models can be used for developing surrogate models for impact loading.

### 3.15 Contact of Articular Cartilage with Implants

The asymptotic model [equations (3)–(6)] covers the case of unilateral frictionless contact between the cartilage layers, and it was applied to study the difference in the contact pressure patterns in the normal and pathological (early stage of osteoarthritis) situations (Wu et al., 2000). In a marginal pathological situation, a part of the diseased cartilage can be replaced by an artificial tissue (Hung et al., 2003; Mano and Reis, 2007) or even with a metallic implant (Manda and Eriksson, 2012). The corresponding contact problems did not receive much attention so far (Hale et al., 1993; Owen and Wayne, 2011; Manda and Eriksson, 2014).

### 3.16 Multiscale Structural Modeling of Articular Cartilage

The asymptotic model [equations (3)–(6)] can be regarded as a “rude” model, since it operates with average constant parameters and does not reflect the actual microstructure of articular cartilage, which represents an important factor in the pathogenesis of osteoarthritis (Buckwalter et al., 2013). From the point of view of multiscale modeling, this asymptotic model represents a macro-level, and to our knowledge, no link has been established with micro-level modeling framework. A certain progress in this direction was made by Federico et al. (2005) who constructed the TITH model by employing the homogenization scheme developed for fiber-reinforced elastic composite materials. The next step can be made by utilizing the recent theoretical development for poroelastic materials (Hellmich et al., 2009; Ortega et al., 2010).

## 4 Conclusion

The asymptotic models presented and discussed above are generally nowadays regarded as oversimplified mathematical models. By all means, the articular contact mechanics should not be confronted by asymptotic modeling alone. At the same time, asymptotic models can be successfully used to facilitate FEM analysis. It is to emphasize that each asymptotic model yields an approximate solution to a problem under consideration, whose accuracy and robustness can be evaluated when the exact solution (analytical or numerical) is available [see, e.g., the examples of refined contact problem and its asymptotic model studied by Mishuris and Argatov (2009)]. One benefit of using simplified (with a limited number of parameters) models, which by construction preserve essential features of the contact system captured by more complex mathematical models, is that they can be employed for uncertainty quantification, when detailed mechanical and geometrical aspects of the system are not fully known. Simple as they are, such models provide a robust theoretical framework for the preliminary analysis of the experimental data as well as for controlling computer simulations produced on the basis of more complicated mathematical models.

## Author Contributions

IA and GM worked on each part of this paper together.

## Conflict of Interest Statement

The authors declare that the research was conducted in the absence of any commercial or financial relationships that could be construed as a potential conflict of interest.
